# AFB1 Toxicity in Human Food and Animal Feed Consumption: A Review of Experimental Treatments and Preventive Measures

**DOI:** 10.3390/ijms25105305

**Published:** 2024-05-13

**Authors:** Agnieszka Pożarska, Krzysztof Karpiesiuk, Wojciech Kozera, Urszula Czarnik, Michał Dąbrowski, Łukasz Zielonka

**Affiliations:** 1 Department of Pig Breeding, Faculty of Animal Bioengineering, University of Warmia and Mazury in Olsztyn, Oczapowskiego 5, 10-719 Olsztyn, Poland; 2 Department of Veterinary Prevention and Feed Hygiene, Faculty of Veterinary Medicine, University of Warmia and Mazury in Olsztyn, Oczapowskiego 13, 10-718 Olsztyn, Poland

**Keywords:** aflatoxin, *Aspergillus flavus*, animal model, pig

## Abstract

Aims: The current review aims to outline and summarize the latest research on aflatoxin, with research studies describing natural, herbal and chemical compound applications in animal (pig) models and in vitro cellular studies. Aflatoxin, a carcinogenic toxin metabolite, is produced by *Aspergillus flavus* in humid environments, posing a threat to human health and crop production. The current treatment involves the prevention of exposure to aflatoxin and counteracting its harmful toxic effects, enabling survival and research studies on an antidote for aflatoxin. Objectives: To summarize current research prospects and to outline the influence of aflatoxin on animal forage in farm production, food and crop processing. The research application of remedies to treat aflatoxin is undergoing development to pinpoint biochemical pathways responsible for aflatoxin effects transmission and actions of treatment. Significance: To underline the environmental stress of aflatoxin on meat and dairy products; to describe clinical syndromes associated with aflatoxicosis on human health that are counteracted with proposed treatment and preventive interventions. To understand how to improve the health of farm animals with feed conditions.

## 1. Introduction

The mold disease aflatoxicosis was discovered in England in the 1960s when 100,000 turkeys died of an unknown condition called the turkey ‘X’ disease. The symptoms occurred in turkeys following Brazilian groundnut feed meal contaminated with mold toxin *Aspergillus* (*A.*) *flavus* [1].

Secondary metabolites of *A. flavus* include aflatoxin B1 (AFB1) and cyclopiazonic acid, which are known to contaminate many different plant species, e.g., groundnut, cotton seeds, pistachios and corn. Other mold fungi able to synthesize and secrete AFB1 are *A. parasiticus* and *A. nomius*. The whole genome of *A. flavus* has been sequenced recently [2]. Storage crop fungi, such as those that produce AFB1, spread due to inadequate ventilation and mishandling of the goods and are a serious risk to agriculture and animal feed production. AFB1 is a lipid-soluble agent that is synthesized optimally at 25 °C and is a derivative of difuranoxano-naphtho-ketone consisting of a difuran ring and a coumarin ring, is carcinogenic, and there are over 20 AFB described to this day, e.g., B2, B2a, G1, G2a, and M2. The allowed limits for AFB1 presence in feed for pigs are 20 µg/kg for feed materials, 10 µg/kg for complementary and complete feed materials, 20 µg/kg compound feed for pigs, and 5 µg/kg compound feed for piglets, being the strictest recommendation limits out of all mycotoxins found in pig feed [3].

Cows metabolize AFB1 to AFM1 by hepatic CYP450 biotransformation, and the AFM1 molecule that is soluble in water is secreted with milk around 5 h after AFB1 consumption at the efficiency of 1–3%. AFM1 is ten times less carcinogenic than AFB1 by international classification for cancer. It was proven that the daily allowance of AFB1 in cow’s feed cannot be higher than 3.4 µg/kg of AFB1 to meet the allowed AFM1 limit of 0.05 µg/L in milk [4]. In human consumers of cereal products, 199 subjects out of 444 tested positive for AFM1 in urine samples, with 37 exceeding the limit for detection of 0.64 ng/mL [5]. There is no official antidote for AFB1 poisoning approved by the Food and Drug Administration (FDA), and the current methods of remediation involve the elimination of the aflatoxicosis symptoms, where the moderately affected individuals, human or animals, have the ability to recover and the main precaution at present is the prevention of exposure to AFB1 [6].

The environmental climate is a factor in mold growth. Brazil experiences a damp environment most of the year where conductance of the mold is throughout and prevention of contamination is insufficient due to agriculture and farming practices, as in many other parts of the world [7]. *A. flavus* appears naturally in the soil and can contaminate food crops to the harvest and post-harvest stages. A study of Brazilian groundnut samples indicated that 14% of the samples exceeded the accepted limit of 20 µg/kg of AFB1 [8]. Investigation of baby food products indicated that 23% of baby food worldwide is contaminated with aflatoxins [9]. Other sources of exposure to AFB1 are workplaces, e.g., furniture workers displayed elevated concentration levels of blood complexes of AFB1/albumin (AFB1/alb) at 0.10 ng/g AFB1/alb compared to levels of 0.06 ng/g AFB1/alb in controls (*p*-value < 0.0001). The workers exposed to wood dust had increased liver enzymes aspartate transferase (AST), alanine transferase (ALT), malondialdehyde (MDA), glutathione peroxidase (GPx), oxidative stress markers and a marked inverse correlation with catalase (CAT) being an example of the common exposure of humans to AFB1 coming from wood sources and its consequences [10]. Hospital patients with prior elevation of ALT enzymes of an unknown etiology were positive for AFB1 presence in 38 out of 50 patients, with a direct positive association between AFB1 and ALT [11].

**Figure 1:**
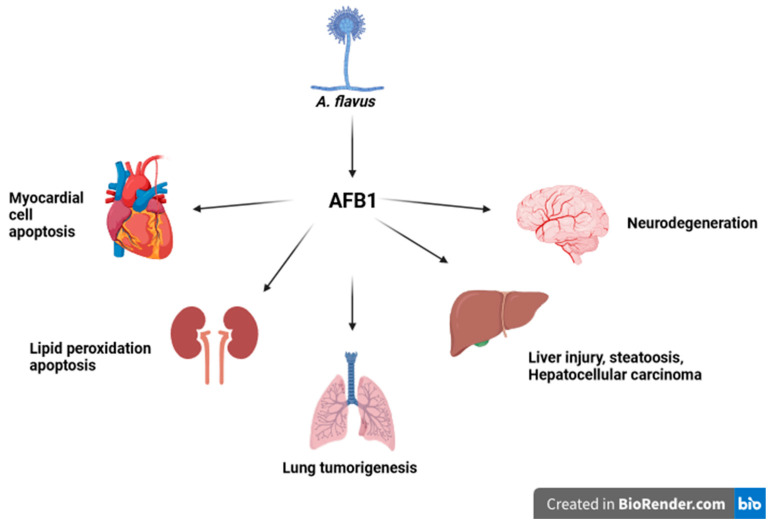
Origins of AFB1 and its effect on different body organs. *A. flavus* synthesizes AFB1, which impacts different body organs and causes injury.

Molecular actions caused by AFB1 are under investigation, and the symptoms described of AFB1 ingestion and exposure list, among others, induction of carcinogenic oxidative damage mechanism [12], premature aging [13], damage to the intestinal barrier protein junctions [14], injury to developing fetuses [15], the heightened risks of infectious diseases [16], and other symptoms (Figure 1). Currently, a novel topic of interest in AFB1 research includes the effects of AFB1 on MMP1 and MMP7 in the liver that could reveal a map of connections of architecture that cause liver injury [17]. The increasing prevalence of AFB1 toxic effects results in the research exploiting possibilities for treatment and prevention of AFB1 poisoning, which is leading to the search for long-term and large-scale solutions involving the use of the environmental data, pharmaceutical, natural and/or dietary approaches using various animal models applied due to the relevance in agriculture and human food production.

## 2. AFB1 Prevalence, Detection, and Treatment of the AFB1 Animal Model

Secondary metabolites of *A. flavus* appear regularly in humid environments. The relation between the growth of molds and storage conditions was exploited with Brazil nuts inoculated with *A. nominus* together with AFB1. AFB1 levels were elevated in 55 out of 78 samples of Brazil nuts, which exceeded the limit of 4 µg/kg. The amount of fungi increased due to humidity, with the highest levels of AFB1 synthesized at 97% humidity. The high levels of storage humidity reflect the relevant results of the average climate in Brazil and could model the spread of aflatoxigenic fungi in the sub-tropical environment [18]. Aflatoxicosis can develop before harvest when drought occurs and causes injury to the seeds. In the study of the different time-points of harvest of groundnuts under drought conditions, the influence of the presence of AFB1 during different harvest time points, with time-points researched being the ‘ideal harvest time’, the ‘harvest from two weeks earlier-’, and ‘the harvest from one week earlier- than the ideal harvest time’. The samples from three different time points differed in AFB1 concentrations in peanut kernels, with the one ‘week earlier than the ideal harvest time point’ concentration at the level of 9.9 µg/kg compared to 614 µg/kg of AFB1 in ‘the ideal harvest time’. These results could indicate that the amount of AFB1 increases exponentially in the last weeks of the groundnut ripening under drought pressure conditions, and a change to the earlier time points of the harvest could decrease the amount of AFB1 present [19].

**Figure 2:**
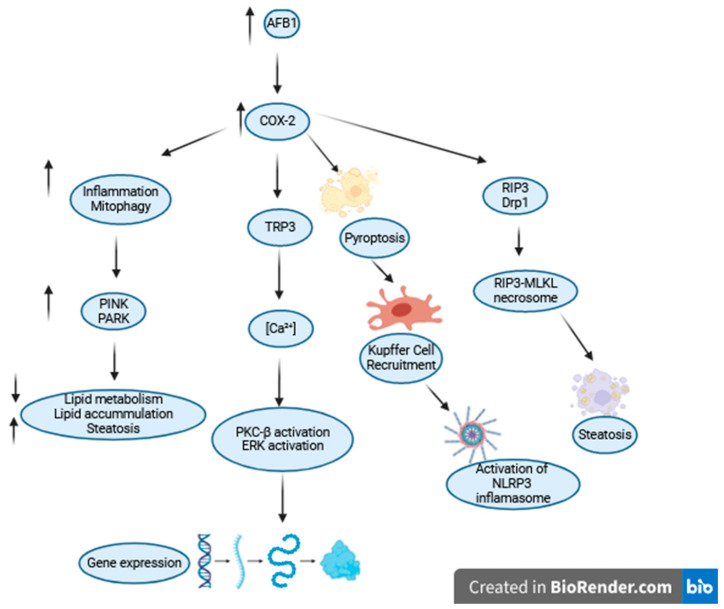
Changes induced by the COX2 enzyme following AFB1 ingestion include cellular inflammation, mitophagy, PKCβ-ERK pathway activation, pyroptosis, and steatosis.

Further effects of AFB1 on health involve pathogenies to fertility by changing the hormone levels, with a decrease in the levels of luteinizing hormone (LH) and upregulation of follicle-stimulating hormone (FSH), progesterone, and hepatic alpha-fetoprotein (AFP) in humans. This can result in damage to soft tissue development, internal hydrocephalus, microphthalmia, immune changes and modifications to liver lobes [20]. The correct cellular machinery organization of the organelles leads to cellular organized metabolism. Exposure of 200 µM of AFB1 in ICR mouse oocytes resulted in 60% of mitochondria being diffused, leading to a faulty mitochondrial respiratory chain within the cell, in comparison to 20% of misplaced mitochondria in the control group [21]. To provide medical measures for AFB1 poisoning, a range of preventive methods and medicinal substances have been tested in research studies. The application of 400 mg/kg of curcumin in feed to ducks for 21 days to alleviate mycotoxin and AFB1 actions with amelioration of the effects on cell death and increase in oxidative stress was suggested in the study that prevented cell death and nephrotoxicity in ducks [22]. AFB1 was affirmed to induce COX-2 expression in a dose-dependent manner in vivo and in vitro in HepG2 and JEG-3 cells [23]. Activation of COX2 leads to transient receptor potential channels (TRPC)-3 activation and a rise in the intracellular cancer levels, activating PKC-β and ERK signal transduction [24]. The induction of COX-2 in a study in mice and HepG2 cells by AFB1 led to increased inflammation and mitophagy, causing liver injury and steatosis. The experimental knockout of the COX-2 enzyme using CRISPR-Cas9 alleviated the toxic effects in HepG2 cells and confirmed AFB1 direct mechanisms on the lipid droplet metabolism in the mitochondria [23]. In another report, AFB1-activated apoptosis led to dephosphorylation of COX-2 by protein phosphatase 2A (PP2a), which caused the recruitment of Kupffer cells in C57BL/6 mice and HepaRG cells. As a result, there was an increase and activation of NLRP3 inflammasome complex both in vivo and in vitro, caspase cleavage, and IL-1β release that regulate liver inflammatory injury [25]. CRISPR/Cas9 induced knockout of COX-2 in HepG2 cells resulted in alleviation of AFB1-induced steatosis and mitophagy. Inhibition of PTEN-induced putative kinase decreasedFB1-mediated liver lipid injury-steatosis [23]. Activation of COX-2 by AFB1 together with HBx mediates receptor interaction protein (RIP3) and dynamin-related protein (Drp1) complex recruitment, which induces liver steatosis [26]. In summary, COX-2 plays an important role in AFB1-induced inflammatory changes in different cell types, adjusts gene expression and releases pro-inflammatory mediators (Figure 2).

Bacterial cell wall peptidoglycan isolated from *L. reuteri* was measured to be composed of 60.49% protein and 37.26% D-glucose poly-chain and, when tested, displayed activity against AFB1 tested in hyaline brown broiler chicks for 42 days. Chicks were fed an AFB1 diet that altered their health status in terms of deterioration of feed intake, blood cell indicators, immunosuppression, and liver steatosis. *L. reuteri* peptidoglycan supplementation resulted in 64.3–75.9% improvement of absorption efficiency of AFB1, improvement of the average gain, feed conversion ratio, white blood cells count, hemoglobin content, GPx activity, immunoglobulin (Ig) A, IgG, IgM and liver changes; weights of immune organs, and NDV-Ab, IBDV-Ab titers [27]. Detoxification of AFB1 is performed by phase I and phase II metabolizing enzymes in liver cytosol and microsomes in the bio-activation and detoxification steps mediated by varied hepatic detoxification cytochromes enzymes. In humans, cytochrome (CYP) P450, CYP1A2, CYP3A4, CYP3A5, and CYP3A7 in the liver and CYP2A13 in the lungs are necessary for bio-activation and metabolism of AFB1 to the reactive electrophilic metabolite exo-AFB1-8,9-epoxide (AFBO). It is estimated that 80% of AFBO can covalently attach to proteins and DNA to form DNA adducts, inducing cellular DNA damage, mutagenesis and carcinogenicity [28].

In humans, AFBO is detoxified by actions of the GSH enzyme metabolic pathway. In the liver, GST addition induces the main detox pathway by binding reactive electrophilic molecules. GST substrates include epoxides of AFB1 acrylamides. Epoxidation-2,3 of AFB1 by CYP450 causes the production of carcinogens in one possible metabolic biochemical route. To improve biotransformation, several studies explored the addition of external bile acids that proved to protect chickens from AFB1-induced liver abnormalities and increased transformation, which leads to the affirmed excretion of the gallbladder and cecum [29]. Elimination of AFB1 was improved by 64% when cell wall fractions of B. thuringiensis AMK10/1 were added, and the effects were due to the properties of the surface layer fraction of the cell wall [30]. Taurine, a sulfur-containing amino acid involved in cell volume regulation and bile salt production, was shown to counteract oxidative stress and mitochondria-mediated apoptosis induced in the liver by AFB1 in the rat [31].

An effective herbal remedy to counter AFB1-induced heightened lipid peroxidation of the cell was modified Siddha extract Kalpaamruthaa (KA), which reversed the effects of AFB1 toxicity on lipid peroxidation and enzyme levels to the control values [32]. Natural flavonol morin occurring in fruits and plants was tested in rats to ameliorate the effects of AFB1. Morin addition to an AFB1-containing diet resulted in significantly improved cardiac and hepatic indicators of malondialdehyde (MDA), GSH, GSH-Px, and the antioxidant enzymes superoxide dismutase (SOD) and CAT [33].

Pigs are farm animals that provide a major meat source and fulfil nutrition needs [34]. The effects of aflatoxin consumption on swine are diminished growth, feed efficiency, jaundice and inhibition of cellular proliferation. While it is estimated that 25% of the pig feed is contaminated with mycotoxins worldwide, pig metabolism is not able to detoxify, eliminate or excrete aflatoxin sufficiently [16]. In a study of a range of AFB1 doses, exposure to the low doses, e.g., 30 µg/kg restricted the growth of Hybrid Grouper, while large doses, e.g., 445 µg/kg, inhibited digestive enzymes, resulted in ROS production and heightened intestinal permeability [35]. To detect the presence of mycotoxins, samples in piggeries are usually taken from feed [36], feces, and blood and for toxicological and mycotoxicological tests. To model and show AFB1 effects in pig breeding, Landrace x Yorkshire gilts received an AFB1 diet of moldy corn (MC) and moldy wheat bran with AFB1 levels of 2.4 µg/kg in 25% MC to 8.9 µg/kg of AFB1 in 100% MC diet t. As a result, there was a decrease in the average daily gain, serum concentration of insulin-like growth factor 1 (IGF-1), growth signaling and swine reproduction [37]. In another study to treat the effects of AFB1, grape seed extract was applied in an 8% meal content to pigs, and the results presented the ability of grape seed extract to detoxify the AFB1 present in the diet (320 ppb) by an increase in antioxidant enzymes activity, a decrease in the concentration of cytokines and TBARS, and an improvement in growth levels. Another study analyzed piglet livers following a diet with AFB1, grape seed extract and sea buckthorn oil. As a result, diet supplementation improved the structural morphology of the liver and kidney and CYP2E1 mRNA expression, allowing for better bioexcretion of AFB [38]. The use of grape waste in another study was proven to counteract the effects of AFB1 by a decrease in the pro-inflammatory cytokines and TBARS levels in pigs with the AFB1 application [39].

In another study on pigs, AFB1 feed intake resulted in diminished growth rate, lowered feed intake and increased liver enzyme levels. The applied treatment in the study included a blend of anti-mycotoxin components containing saccharomyces cerevisiae lysate, zeolite, silicon dioxide, propylene glycol, *Carduus marianus* extract, soy lecithin, and carbonate. The applied anti-mycotoxin blend improved the overall pig health status, normalized liver enzymes and protected against AFB1 in feed [40]. A study was conducted to assess the hepato-protective effect of silymarin and lupeol, which is a new treatment made from the stem bark of *Crataeva nurvala* extract. The study found that lupeol showed improvement in hepatic damage and decreased the levels of lipid peroxide. The authors concluded that lupeol is an effective agent in protection against AFB1, similar to silymarin in the rat model [41]. In a different study, curcumin and phenolic extract from grapes-resveratrol were assayed in a rat model for 90 days, and curcumin demonstrated a reversal of the pathological serum enzyme biochemical profile and liver histopathological features changes following AFB1 poisoning in male Fischer rats, compared to resveratrol that did not have any positive therapeutic effects in the conducted research [42].

The feed supplements in a study of adult gilts following the addition of AFB1 included treatment with clay, dried yeasts, and yeast culture, which addition resulted in the improvement in monocyte numbers and in the liver bile duct hyperplasia [43]. Porcine kidney cells were CRISPR/Cas9-knocked out and demonstrated that the transcription factors BTB and CNC homolog 1 (BACH1) are necessary to transduce the toxic actions of AFB1. The inhibition of BACH1 in porcine kidney cells and human hepatoma cells resulted in the boosted resistance to AFB1 and alleviation of oxidative damage. The functional screen identified an inhibitor to BACH1, called 1-Piperazineethanol, α-[(1,3-benzodioxol-5-yloxy)methyl]-4-(2-methoxyphenyl) (M2), whose administration improved weight loss and liver injury in vivo in pigs [44]. The hepato-protective potential of another beneficial substance was confirmed when vegetable choline was added to counteract AFB1 in pig feed to alleviate liver damage at a dose of 800 mg/kg applied to the feed base mix [45].

## 3. AFB1 in Animal Injury Models: Dosing and the Use of the Model

After treatment with AFB1, reactive oxygen species (ROS) are produced that harm the liver cells, such as hepatocytes and liver epithelial cells. The AFB1-activated biotransformation generates ROS, which leads to an increase in the liver enzymes, as well as histopathological changes of vacuolar degradation and necrosis of the hepatocytes. The number of vacuolar degenerative cells/mm2 in hepatocytes increased significantly after AFB1 liver toxicity in 6-week-old male Wistar rats, with 747.8 ± 229.7 cells/mm^2^ compared to 12.3 ± 15.9 cells/mm^2^ in the control group [46]. Chemo-protective agents to prevent aflatoxicosis with prior pre-treatment application tested in human hepatocyte cells were oltipraz, sulforaphane, and phenethyl isothiocyanate that acted via actions of possible transcriptional repression of genes that are responsible for AFB bioactivation [47]. 

The mycelial growth, hyphal spread and sprouting of *A. flavus* inhibition occurred on the application of L-Cysteine hydrochloride (L-CH) compound. The inhibition was mediated by genetic expression alteration of genes responsible for the control of the fungi cell wall structure [48]. Plant herbal properties of AFB1 detoxification were assessed for 21 herbal molecules with *Silybum marianum*, *Satureja khuzistanica* and *Moringa oleifera* Lam. and other phytochemicals that improved AFB1 in chicken broiler by modulating apoptosis and pro-inflammatory mechanisms [43]. In the study of the hepato-protective and phyto-antioxidant activity of the sea buckthorn (*Hippophae rhamnoides*) in broiler chicken following toxicity engendered by AFB1, the hepato-damage was alleviated with the application of sea buckthorn oil extract and the levels of COX2, Bcl-2, and p53 were lowered, along with the concentration of AFB1 that decreased from 460.92 ± 6.2 ng/mL in the AFB1 group to 15.59 ± 6.1 ng/mL in the treatment group [44]. After pre-treatment with red pitaya fruit juice extract for 30 days, rats were exposed to 250 µg/kg AFB1 for two days. The study found that the juice extract prevented the toxic symptoms of AFB1. This was demonstrated by the normalization of ALP concentration and the reduction of noxious effects on TBARS, RS, Hsp-70, CAT, GSH, and Nrf-2. Moreover, the juice extract also prevented oxidative damage [45]. Chicken feed enrichment with phytobiotic and toxin binders feed had remediated growth performance, biochemical blood markers and intestinal morphology in broiler chickens [49]. A study was conducted on turkey poults using an adsorbent material made of powdered alfalfa leaves. This material was applied to a diet contaminated with AFB-1. The study found that the use of this material resulted in an improvement in total protein, glucose, calcium, creatinine, and blood urea nitrogen levels. This, in turn, led to an increase in body weight and feed intake. However, no significant changes were observed in blood count or liver enzymes [47].

Turmeric powder was proven to relieve AFB1-induced toxicity in broiler chicken, increase the expression of CYP2A6 mRNA transcript, and reduce the expression of NF-E2-related nuclear factor 2 (Nrf-2) transcript. The expression of AFB1 was decreased by turmeric powder, by mechanisms including enhancement of the expression of ABCG2 mRNA in the duodenum [50]. AFB1 is known to cause damage to the immune system and spleen, and the protective role of the *Penthorum chinese* Pursh compound (PCPC) in this condition was reported to be preventive and medicinal acting via the JAK/STAT pathway of cell death in the spleen of broiler chicken [51]. In a different study, it was found that AFB1 causes neurotoxicity by impacting the endoplasmic reticulum (ER) stress in C57BL/6J mice and HT22 cells. The study also showed that this action involves the GSH/GST enzyme system and increases the expression of the AHR receptor. However, these toxic observations were reversed with the treatment of flavonoid glycoside hesperetin [52]. Furthermore, the addition of red chili pericarp and seed powder as a feed additive to the day-old broiler chick diet helped negate DNA damage, improve total protein and globulin concentrations, and reinforce liver enzyme levels induced by 0.5 mg/kg AFB1. Red chili powder improved the red blood cell count, packed cell volume, and hemoglobin concentration. AFB1 damage to the bone marrow can hinder erythropoiesis and RBC production. However, these effects can be counteracted using natural nutraceutical ingredients such as red chili powder, which is composed of capsaicinoids and carotenoids [53]. Phytochemical molecules such as luteolin (LUTN) have been proven to counteract the harmful effects of AFB1. In rats, luteolin has been shown to decrease levels of hepatic ALT and AST, MDA and ROS, while improving expression of CAT and SOD. By doing so, luteolin ameliorates oxidative stress [54].

## 4. Novel Development of the Application of Microbiota in AFB1 Toxicity Relief

Exposure to AFB1 following ingestion was demonstrated to change the abundance and diversity of the gut microbiota in rats [55]. The increase in research on the microbiota in counteracting AFB1 toxicity has provided a beneficial source of knowledge for future therapies. Studies have shown that *Rhizoma Drynariae* (TFRD) can improve the expression of intestinal tight junction proteins and promote healthy epithelial structure. This can help regulate gut microbiota and liver functions affected by AFB1 deregulation. Additionally, the growth of beneficial bacteria suggests that herbal treatments like TFRD can be useful in treating aflatoxicosis [56].

An endophytic bacterium with the identifier MS455 was isolated from soybean and exhibited a broad-spectrum anti-fungal activity. Genomic analysis revealed that it belongs to the Burkholderia genus and shares high homology with ocf gene clusters, which produce an antifungal compound called occidiofungin, a process regulated by the ORF1 gene. MS455 was shown to inhibit the growth of *A. flavus* on plate colonies assay, which could contribute to the development of novel methods of inhibition of *A. flavus* growth in plant production [57]. Lactic acid bacteria (LAB) isolates and supernatants were tested to mitigate AFB1 with a streak plate method and measurement of the inhibition zone formed around wells followed by 16S sequencing of rDNA, and it was shown that LA.12 Lactobacillus harbinesis 487 resulted in the best possibility for detoxification of AFB1 and application in corn.

The fecal microbiomes of sheep were studied, and the results suggested elevation in *Bifidobacterium* bacterium as a probable biomarker for AFB1 presence [58]. Aflatoxin was discovered to be present accompanied by a range of other mycotoxins, e.g., ochratoxins A and B, zearalenone, deoxynivalenol, and sterigmatocystin in feed of agriculture animals with the highest amount of AFB1 assayed in poultry feed at 6.9 µg/kg, and 887 µg/kg of deoxynivalenol toxins assayed in sheep feed in Spain [59]. A probiotic composed of Lactobacillus plantarum reduced AFB1 concentration in the feed, and a study of chitosan coating prophylactic potency of *Lactobacillus plantarum* RM1 nano-emulsion (CS-RM1) in rats fed with AFM1 proved reversed toxicological parameters and markedly improved the morphology of liver and kidneys. The inclusion of CS-RM1 as a supplement to milk to eliminate the harmful presence of AFM1 was proven to be beneficial [60] The level of AFB1 elimination was proven to be 64% achievable using gram-positive microbes Firmicutes, measured by cell wall fractions. The surface layer of cell fraction (S-layer) played a crucial role in the elimination process [61].

In the study of adsorbents, i.e., organically modified clinoptilolite (OMC) and multi-component mycotoxin detoxifying agent (MMDA), the AFB1 fed-piglets displayed a reduction in the incidence of liver cancer without any qualitative deleterious actions [62]. A study was conducted to investigate the effects of a spray-dried porcine plasma high-quality protein as a functional food ingredient for piglets to protect them against AFB1. The study found that piglets who had this protein incorporated into their diet showed improvements in growth performance, ROS levels, and body mass. Additionally, there were no changes observed in the antioxidant enzyme CAT [63].

New sequestering agents (SA) were tested in cow feed to measure the ability to decrease the concentration of aflatoxin, with the application of the SA to the feed resulting in the reduction of AFM1 concentration from 0.016 µg/kg to 0.008 µg/kg in milk, with no changes to cow health or reproduction status [64] Studies of various bioactive methanolic plant extracts explored the plants’ phytochemical activities against AFB1. Screened substances demonstrated a time-dependent AFB1 detoxification capability that ranged from 20.17% to 38.13%, with the highest anti-mycotoxin activity reaching 40.81% [65]. The traditional Chinese herbal medicine plant *Hedyotis diffusa* was tested for AFB1 actions on the liver and proved to decrease hepatic pathological alterations of ALT, AST, ALP, TP, and ALB, and reduce DNA-adduct formation in the liver by mechanisms involving an increase in the expression Nrf2, and heme oxygenase 1 (HO-1) [66]. In another study, three herbal extracts *Ixora coccinea* (Rubiaceae), *Rhinacanthus nasuta* (Acanthaceae), and whole plants of *Spilanthes ciliata* (Asteraceae), were tested on albino Wistar rats. The findings included protection against AFB1-induced liver damage demonstrated by decreased serum enzyme activities and improved liver GSH status [67]. Further details of studies of AFB1 models and treatments are summarized in Table 1.

## 5. Biomedical Development to Prevent AFB1 Poisoning

The test of the efficacy of different mycotoxin binding (MTB) agents of AFM1 in milk, including hydrated sodium aluminosilicate (HSCAS), yeast cell wall (YCW), and bentonite, resulted in a decrease in the concentration of AFM1 detected in milk [75]. Another new treatment proposed involved berberine-loaded albumin nanoparticles (BER-BSA NP) that displayed improved bioavailability as an anticancer drug with a small hydrodynamic size of nanoparticles (320 ± 13.2 nm). The application of BER-BSA NP countered the expression of AFP, liver vacuolar and hydropic degeneration and decreased levels of necrotic hepatocytes [76].

Hexanal was tested to cause early apoptosis of *A. flavus* conidia by mechanisms involving mitochondrial function disruption and altering the expression of key energy genes, showing promising value for use in farming applications in the form of a vapor spray that inhibits the growth and spread of *A. flavus* on wheat grains crops [77]. Inhibition of the expression of AFB1 activated gene cluster, 2-isopropyl benzaldehyde thiosemicarbazone (mHtcum), was applied to inhibit the gene synthesis in *A. flavus*. The functional screen in the yeast was performed to identify molecular mechanisms by which mHtcum exhibited inhibitory actions, and as a result, the main pathway discovered to be altered by mHtcum were mitochondrial respiratory chain genes COR1 and COR2 [78].

Mycotoxin detoxifier compound (CMD) was analyzed in broiler chicken and was found to improve the toxic effects of the growth. It also showed anti-inflammatory effects and improved the histology, growth rate, and organ weight of the thymus. The composition of gut chicken microbiota was measured on AFB1 addition, and AFB-induced microbiota changes were reversed to the basal levels after MD addition to the chicken feed [79]. To test the impact of various variations of chicken feed on AFB1 impact, the samples from pelleted poultry feed from 84 samples randomly collected from chicken feeding stalls in Iran were measured to contain levels of AFB1s ranging up to 5.58 µg/kg and the AFB1 levels in mash feed were measured at t 3.31 ppb [80].

The pre-treatment with Eucalyptus oil (EO) extracted from the Eucalyptus plant was applied in rats for 14 days, followed by two doses of AFB1 protected against biochemical changes such as myeloperoxidase (MPO) and hydrogen peroxide activity and reduction in antioxidant enzymes GPx and SOD and improved condition of the gastric and intestinal mucosa. The main active ingredient of EO is 1, 8-cineole applied as a control, which does not induce any changes in rats [81].

## 6. Hepatocarcinoma with Respect to AFB1 Toxicity

**Figure 3:**
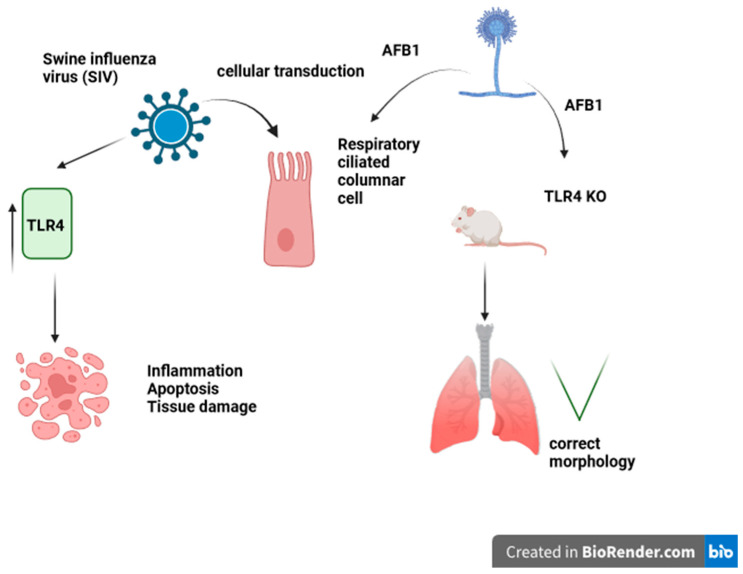
Swine Influenza virus (SIV)–induced activation of respiratory epithelium apoptosis. Toll-like receptor 4 (TLR4) KO mice are protected against AFB1-induced lung damage.

Liver cancer is one of the leading causes of death worldwide, and AFB1 poisoning is considered a significant non-genetic factor contributing to the induction of liver cancer hepatocarcinoma (HCC). Mechanistic synergistic effects of induction of cancer were demonstrated for AFB1 when AFB1 served as a co-factor in the induction of HCC in combination with HBV virus and Epstein-Barr virus (EBV) in a review of animal studies of viral and AFB1 combined pathological outcomes. The AFB1 was able to increase the efficiency of EBV infection in primary B cells via an increase in DNA replication [82]. Other studies have determined that AFB1 is able to enhance both EBV and influenza virus severity and acts as a co-factor for EBV HBV carcinogenicity [83]. In other genome-wide association studies (GWAS), the importance of hereditary conditions contributing to the viral and environmental risk factors was presented. The genome-wide CRISPR-Cas9 genetic screen in PLC/PRF/5 cells was performed to identify AFB1-induced genetic changes in hepatocytes, with the main genetic alterations outlined being the increase in expression of the AHR receptor, which causes induction of the expression of P450. The expression of AHR was also proved to be elevated in tumor sections from AFB1-HCC patients and positively correlated with the presence of increased levels of the immune regulator PD-L1 [84]. In order to find new treatments for AFB1-induced toxicity and HCC, it is necessary to develop and apply appropriate models in research. To simulate and induce HCC, a single i.p. injection of AFB1 was administered to rats, which was found to be effective in inducing the pathological changes associated with HCC. It was demonstrated that Glycine N-methyltransferase (GNMT) is downregulated in HCC, and the overexpression of GNMT results in the prevention of AFB1-induced carcinogenicity, together with downgraded liver cancer cell proliferation. A drug screening platform used a GNMT promoter-driven luciferase reported assay to identify *Paeonia lactiflora* Pall (PL) extract and the active ingredient 1,2,3,4,6-penta-O-galloyl-β-D-glucopyranoside (PGG) as GNMT activator molecules. It was confirmed that both PL and PGG could induce GNMT expression in Huh7 human hepatoma cells and in xenograft tumors. PGG treatment alone induced apoptosis in Huh7 cells, showing the anti-carcinogenic potential of this treatment. Investigating and blocking the effects of AFB1-induced pathological changes can lead to finding effective and important therapeutic agents for the treatment of HCC, which will provide hope for the patients [85]. Swine influenza virus (SIV) belongs to the influenza A subtype, which is enzootic throughout pig farms and can cause lung disease and pandemic-like pig-to-human circulation [86]. A study of the impact of low-level AFB1 doses ranging from 0.01–0.25 μg/mL on SIV replication in vivo in mice demonstrated increases in the rate of SIV replication, a heightened level of expression of viral matrix protein, toll-like receptor 4 (TLR4), viral titer, nucleoprotein, matrix protein 1 (M), the release of tumor necrosis factor (TNF)-α, and an increase in IL-10. Release and interaction of the inflammatory factors combined with the presence of AFB1 promoted lung injury and lethality via interaction with TLR4 [87] (Figure 3).

Biomarkers are proposed and used to test the presence of pathological agents and conditions [88]. Detection of the concentration of blood biomarkers that are prognostic for liver carcinogenesis and disease following AFB1 contamination would aid in clinical diagnosis [89]. Curcumin was shown to improve inflammation and autophagy via Nrf2 pathway following AFB1-containing meal feed in broiler chickens [69]. In a study comparing the effects of a standardized extract of iridoid glycoside fraction of *Picrorhiza kurroa picroliv* to silymarin, both treatments demonstrated holistic effects on enzyme- and hepato-protection in a rat model [70].

Genome-wide association studies (GWAS) proved the importance of hereditary factors contributing to the viral and environmental risks in relation to AFB1 [90]. Phenolics-rich ginger extract (GE) applied at 100 and 250 mg/kg daily inhibited the production of intracellular ROS, DNA strand break, and lipid peroxidation [71]. In Japanese quail, the dose of 1500 µg/kg of AFB1 delivered in six applications was sufficient to produce hepatotoxic results of an increase in blood concentrations of AST, GGT, and CK, and the application of 500 g/ton silymarin in feed did not improve the symptoms [91].

To study the effect of three herbal plants (*N. Sativa*, *P. Ginseng*, and *C. Sempervirens*) in albino Wistar rats, AFB1 was administered to induce carcinoma rat symptoms, and the applied treatment herbal combination reduced IL-6, hs-CRP, and MDA molecule markers [72]. Another study of natural ingredients in rats, i.e., honey in an AFB1-induced model of toxicity, alleviated toxic symptoms, lipid peroxidation and liver enzymes, and increased enzymatic antioxidant expression [73].

AFB1 actions were reported to cause neurological genetic instability resulting in autism-like apoptosis presented in the autism BTBR model characterized by an increase in the micronuclei, oxidative DNA strand break, increased lipid peroxidation, and redox imbalance. The BTBR autism model genetic changes included the impairment of the DNA repair pathway, downregulation of the associated Xrcc1 gene and an increase in the Gadd45a gene responsible for mitochondrial loss and muscle weakness. Further genetic alterations include mutations in DNA repair genes p53 and increased the apoptotic number of bone marrow cells [92]. In a mouse model, ingestion of AFB1 resulted in the accumulation of AFB1 in the brain without the presence of metabolite AFBO, causing brain injury. This led to downregulation of the DNA repair gene RAD51 and decreased cell viability [93]. In a rat neurodegenerative study, AFB1 was added to feed at doses of 0.75 and 1.5 mg/kg for 30 days, resulting in neurodegenerative damage characterized by a decrease in acetylcholinesterase activity, SOD and CAT expression in the hippocampus, and an increase in inflammatory mediators IL-6, nitric oxide (NO), and myeloperoxidase (MPO). Rutin, a glycoside flavonol compound with a molecular weight of 610.5 g/mol, was applied as a treatment at a dose of 50 mg/kg in feed. It displayed anti-neurodegenerative effects, improved the cerebrocortex condition, activated molecular pathways that corrected IL-6, NO, and MPO expression levels, reduced oxidative stress markers, and increased hydrolysis of the purinergic molecules in the brain areas (Table 1) [74].

The further disturbance of AFB1 to genetics and development was presented in zebrafish when liver development was altered, and fat accumulation was present in the liver. Transcriptomic sequencing results presented alterations in liver oxidative metabolism and genetic pathways of developing larvae. A KEGG analysis indicated that AFB1 addition resulted in main genetic changes that included the glutathione enzyme genetic pathway [94].

Training and application of wheat, maize, and other crops post-harvest techniques among farmers resulted in a 53% decrease in AFB1 occurrence in maize, which is attributed to drying crops in rural areas with drying sheets. In another study method, the use of detoxification measures post-harvest reduced AFB1 content by 80% after 1 h of application in red peppers. Further improvements for application at the post-harvest stage to reduce AFB1 contamination included the drying of crops and the use of special bags in storage. Novel developments in AFB1 detection for reporting involve the development of cascade amplification detectors [95] and biosensor detectors [96]. Neutral electrolyzed water was applied to detoxify aflatoxins with application to AFB1 and AFB2-containing turkey feed maize. The neutralized maize was tested in turkeys, and as a result, the amelioration of AFB1 and AFB2 occurred, assayed by biochemical blood measurements, enzyme activities and organ morphology [97].

AFB1 research involves characterization of the induced AFB1 toxicity to create an adequate model to reflect a clinical image, the influence of AFB1 on the body’s chemical composition, metabolic pathways alterations, preventive treatments, and characterization of the current presence in the environment. This research is necessary to counteract and mitigate the spread of *A. flavus* and AFB1 poisoning. This should be coupled with sufficient detection of AFB1 in food products and animal feed imported from developing countries. The remaining questions of ethics and practical areas remain whether animals or humans should have access to preventive medicines to prevent the harmful effects of AFB1s, considering the high incidence of HCC and its effects on the health of animals.

AFB1 is a global health threat that causes cancer and human and animal feed poisoning. It is an agent that is not yet fully controlled. Studies have shown that there are agents and treatments available to counteract AFB1, as well as novel methods of detection and measures to protect farm crops and the environment from AFB1 contamination. There are preparations already in use that prevent fungi growth and neutralize fungi spread. While there are still no definite agents that could prevent AFB1 liver cancer pathogenesis, a number of studies have described the positive preventive effects of herbs and other remedial and microbial measures. The development of methods to eliminate AFB1 from animal feed, improve storage conditions, and improve food quality and agriculture safety and prosperity is ongoing.

## Figures and Tables

**Table 1:** Summary of studies describing the AFB1 model and treatment.

Treatment, Study Ref., Study Title	AFB1 Dose, Route Application, Species, Time of Treatment	Characteristics	Results
Curcumin 400 mg/kg, feed [22]; Curcumin alleviates AFB1-induced nephrotoxicity in ducks: regulating mitochondrial oxidative stress, ferritinophagy, and ferroptosis.	0.1 mg/kg bw to ducks; gavage for 21 days	Increased mitochondrial oxidative stress, increase in MDA, 8-OHdG	Improves nephrotoxicity by inhibiting mitochondrial oxidative stress
Peptidoglycan *L. reuteri* 200 mg/kg/feed [27]; *Limosilactobacillus reuteri* peptidoglycan alleviates aflatoxin B1-induced toxicity through adsorbing toxins and improving growth, antioxidant status, immunity and liver pathological changes in chicks.	71.43 µg/kg in Hy-Line brown chicks, feed for 42 days	Increased the plasma MDA content and decreased SOD and GSH-Px activity and T-AOC	Toxin adsorption, Improved immunotoxicity and hepatic status
Morin 30 mg/kg bw, oral [33]; Acute aflatoxin B1-induced hepatic and cardiac oxidative damage in rats: Ameliorative effects of morin.	2.5 mg/kg bw to Wistar Albino rats, oral, twice on days 12 and 14, during a 15-day experiment	Increase in AST, ALP, LDH, GGT, CK, CK-MB, 8-OHdG, IL-1β, IL-6, TNF-a	Cardiac and hepatic levels improved of MDA, GSH, GSH-Px, antioxidant enzymes
Grape seed extract (320 ppm) [38]; Effectiveness of dietary byproduct antioxidants on induced CYP genes expression and histological alteration in piglets liver and kidney fed with aflatoxin B1 and ochratoxin A.	62 ppb AFB1 to crossbred pigs (TOPIG)-40 hybrid piglets, feed for 30 days	Decrease in antioxidant activity, concentration of cytokines and TBARS	Improvement in structural morphology of liver and kidney
Grape waste feed (8% feed) [39]; Assessment of the efficacy of grape seed extract in counteracting the changes induced by aflatoxin B1 contaminated diet on performance, plasma, liver and intestinal tissues of pigs after weaning.	320 ppb AFB to crossbred pig TOPIG, feed for 30 days	Increase in the pro-inflammatory levels in the liver and colon	Decrease in pro-inflammatory cytokines and TBARS
Lupeol isolate from *Crataeva nurvala*; 100 mg/kg, bw [41]; Lupeol ameliorates aflatoxin B1-induced peroxidative hepatic damage in rats.	1 mg/kg body mass, orally to rats, for 7 days	Increase in the lipid peroxide levels decrease in the enzymatic and non-enzymatic antioxidants	Reversal of hepatic damage and improvement in lipid peroxide levels
Curcumin 200 mg/kg bw; Resveratrol 10 mg/kg bw [42]; Comparative effects of curcumin and resveratrol on aflatoxin B(1)-induced liver injury in rats.	25 µg/kg bw, oral gavage, male Fischer rat for 90 days	Increased liver focal necrosis, liver enzymes, ALT, AST, γ-GT, lipid peroxidation, decrease in GSH, SOD, CAT, GSH-Px	Decrease in ALT, AST, γ-GT, GSH, SOD, CAT, GSH-Px, improved liver histology
Clay 2 mg/kg, dried yeast 1.5 mg/kg, yeast culture 1.1 mg/kg, [43]; The use of feed additives to reduce the effects of aflatoxin and deoxynivalenol on pig growth, organ health and immune status during chronic exposure.	150 µg/kg to gilts, feed for 42 days	Alteration to the immune system through an increase in monocytes and immunoglobulins	Improvement in monocyte numbers, liver duct cellular hyperplasia
Vegetable biocholine 800 mg/kg [45]; Can the inclusion of a vegetable biocholine additive in pig feed contaminated with aflatoxin reduce toxicological impacts on animal health and performance?	500 µg/kg daily to pig; feed for 20 days	Reduced feed consumption and weight gain Increase in the intestinal oxidative markers	Hepatoprotection
Curcumin 300 mg/kg; [49]; Curcumin protects against Aflatoxin B1-induced liver injury in broilers via the modulation of long non-coding RNA expression.	1 mg/kg to broiler chicken; feed; for 28 days	Hepatic injury Increased production of ROS, antioxidant enzymes	Improved morphology, regulation of LncRNAs
Sea buckthorn berries oil 0.6 mL oil/kg of bw/day [68]; The hepatoprotective effect of sea buckthorn (*Hippophae rhamnoides*) berries on induced aflatoxin B1 poisoning in chickens.	54 µg/kg/day to broiler chicken; oral gavage for 28 days	Reduced albumin, Increase in AST	Reduction in liver necrosis and fatty deposits
Turmeric Powder 400 mg/kg [50]; Turmeric powder counteracts oxidative stress and reduces AFB1 content in the liver of broilers exposed to the EU maximum levels of the mycotoxin.	0.02 mg/kg in feed, broiler chicken for 10 days	Increase in lipid peroxidation	Increase in liver gene expression and counteracted lipid peroxidation
Curcumin 450 mg/kg [69]; Curcumin mitigates oxidative damage in broiler liver and ileum caused by aflatoxin B1-contaminated feed through Nrf2 Signaling pathway.	5 mg/kg in broiler chicken; feed, for 28 days	Autophagy reduction, inflammation, mTOR increase, beclin-1, ATG, Nrf2, HO-1, dynein decrease	Inflammation, restored of Nrf2 and HO-1 expression, normalized hepatocytes morphology
Picroliv 25 mg/kg bw; Silymarin 20 mg/kg bw; [70]; Long-term effect of aflatoxin B(1) on lipid peroxidation in rat liver and kidney: effect of picroliv and silymarin.	2 mg/kg bw, single i.p. injection, albino Wistar rat for 6 weeks	Increase in lipid peroxide level, decrease in enzymatic antioxidant levels	Reversal of liver peroxide enzyme pathology
Phenolics-rich ginger extract (GE) 100 and 250 mg/kg daily feed [71]; Protective effects of phenolics-rich extract of ginger against Aflatoxin B1-induced oxidative stress and hepatotoxicity.	200 µg/kg in Wistar rat, i.p. alternate days for 28 days	Toxicity of liver damage, serum markers	Inhibition of the production of intracellular ROS, DNA strand break, lipid peroxidation, Increase in expression of Nrf2/HO-1
N. Sativa 500 mg/kg/day, P. Ginseng 250 mg/kg/day, C. Sempervirens 300 mg/kg/day [72]; IL-6 and NFE2L2: A putative role for the hepatoprotective effect of *N. Sativa, P. Ginseng* and *C. Sempervirens* in AFB-1 induced hepatocellular carcinoma in rats.	150 μg/kg/day to Albino Wistar rats; i.p. for 3 days	HCC	Reduction in IL-6, hs-CRP, MDA
Honey 1 mL/kg [73]; Histopathological and biochemical investigations of the protective role of honey in rats with experimental aflatoxicosis.	25 µg/day; oral gavage, Sprague-Dawley rat for 90 days	Marked liver histopathological lesions, Increase in concentrations of AST, GGT, ALT, decrease in bw, decrease in CAT, GR, SOD	Decrease in lipid peroxidation, liver enzymes, increase in enzymatic and non-enzymatic antioxidants. Normalization of histology in liver and kidney
Rutin 50 mg/kg [74]; Aflatoxin B1-induced redox imbalance in the hippocampus and cerebral cortex of male Wistar rats is accompanied by altered cholinergic, indoleaminergic, and purinergic pathways: Abatement by dietary rutin.	0.75 and 1.5 mg/kg bw, feed, male Wistar rat for 30 days	Neurotoxicity, decreases in acetylcholinesterase activity Decreases of SOD and CATin the hippocampus, Increase in IL-6, NO, MPO	Improved histological image of the cerebral cortex, correction of IL-6, NO, MPO levels, reduction in oxidative stress markers, increased hydrolysis of the purinergic molecules in brain areas

bw—body weight; i.p.—intraperitoneal.
